# DNA Damage and Chromatin Rearrangement Work Together to Promote Neurodegeneration

**DOI:** 10.1007/s12035-024-04331-0

**Published:** 2024-07-08

**Authors:** Harman Sharma, Sushma Koirala, Yee Lian Chew, Anna Konopka

**Affiliations:** Flinders University, Adelaide, South Australia

**Keywords:** DNA damage, Chromatin rearrangement, Neurodegeneration, Hallmark proteins

## Abstract

Neurodegenerative diseases have a complex origin and are composed of genetic and environmental factors. Both DNA damage and chromatin rearrangement are important processes that occur under pathological conditions and in neurons functioning properly. While numerous studies have demonstrated the inseparable relationship between DNA damage and chromatin organization, understanding of this relationship, especially in neurodegenerative diseases, requires further study. Interestingly, recent studies revealed that known hallmark proteins involved in neurodegenerative diseases function in both DNA damage and chromatin reorganization, and this review discusses the current knowledge of this relationship. This review focused on hallmark proteins involved in various neurodegenerative diseases, such as the microtubule-associated protein tau, TAR DNA/RNA binding protein 43 (TDP-43), superoxide dismutase 1 (SOD1), fused in sarcoma (FUS), huntingtin (HTT), α-synuclein, and β-amyloid precursor protein (APP). Hence, DNA damage and chromatin rearrangement are associated with disease mechanisms in distinct neurodegenerative diseases. Targeting common modulators of DNA repair and chromatin reorganization may lead to promising therapies for treating neurodegeneration.

## Introduction

The chromatin structure ensures efficient packaging and utilization of nuclear genetic material. Chromatin governs neuronal function through positioning of genes into proper nuclear compartments to allow for their transcription or silencing [1]. By serving as a platform for integrating and storing signals that regulate gene expression, it governs the temporary implementation of genetic programs within cells [2, 3]. These genetic programs include the regulation of synaptic plasticity, the phenomenon responsible for the strength and efficiency of communication between neurons, ensuring learning and memory, brain development and homeostasis, sensory training, and recovery from brain lesions [4].

Furthermore, chromatin structure determines the magnitude of DNA damage. Neurons are prone to aggregation of DNA lesions, including the most detrimental double-stranded breaks (DSBs). This is due to their high metabolism, post-mitotic conditions, and longevity [5, 6]. If DSBs are unrepaired, they can lead to cell death; alternatively, if they are poorly repaired, they can lead to mutagenesis, chromosome rearrangement, or even loss of genetic information [5]. The chromatin structure is also essential for DNA repair because it enables the recognition of DNA breaks by sensing proteins, provides a scaffold for DNA repair proteins, and initiates DNA damage checkpoint signals [7].

Although the interplay between DNA damage and chromatin organization is well known [8–10], this interplay has only started to attract attention in research on neurodegeneration. Over the past several years, hallmark proteins involved in the function of DNA damage and repair in neurodegenerative diseases have been recognized. Interestingly, the same proteins are implicated in chromatin reorganization. Furthermore, DNA damage occurs during aging and is a major risk factor for neurodegenerative diseases [6, 11].

Thus, in this review, we discuss the interplay between chromatin reorganization and DNA damage in neurodegeneration. We focused on hallmark proteins associated with diseases such as amyotrophic lateral sclerosis, Alzheimer’s disease, frontotemporal dementia, Huntington’s disease, and Parkinson’s disease. For detailed discussion about the general relationship between DNA damage and chromatin outside the scope of this review, we direct readers to other comprehensive articles [12–14].

### Overview of the Regulation of Chromatin Structure

Nucleosomes are basic units of chromatin and are composed of DNA wrapped around histone octamers. The histone octamer is composed of two copies each of the histone proteins H2A, H2B, H3, and H4 [5]. Post-translational modifications of histones tightly regulate the degree of chromatin compaction and accessibility of DNA by adjusting the chromatin structure to relatively accessible or inaccessible subdomains depending on the chemical properties of the protein. Two general states of chromatin compaction are defined: heterochromatin and euchromatin.

Within heterochromatin, which is a condensed, transcriptionally inactive type of chromatin, constitutive and facultative subtypes are distinguished [15]. While constitutive heterochromatin is enriched in trimethylated histone H3 at lysine 9 (H3K9me3), facultative heterochromatin is enriched in trimethylated histone H3 at lysine 27 (H3K27me3) [15]. The methylated histones are bound by proteins, which are specific histone code “readers”, and these are associated with effector proteins that provide the functional basis of heterochromatin. Heterochromatin is regulated by histone modification, but it may also be regulated by the phosphorylation associated with histone proteins [15].

Histone acetylation allows DNA to be unwrapped to an “open”, transcriptionally active euchromatin and occurs on multiple lysine residues, most commonly on histones H3 and H4 [16]. Histone acetylation is catalyzed by histone acetyl transferase in the presence of the cofactor acetyl-CoA. This reaction can be reversed through deacetylation by histone deacetylases (HDACs) [17]. In contrast to histone acetylation, which is correlated with chromatin accessibility and transcriptional activity, the effect of histone methylation depends on which residue is modified [16]. Histone methylation is catalyzed by methyltransferases in the presence of methyl groups and may be present at lysine, arginine, or histidine residues. The reaction is reversed by demethylases. Methylation can cause condensation or relaxation of chromatin depending on its position on histones [18, 19]. Importantly, the combination of specific types and positions of histone modifications determines active or repressive marks of histones, which govern chromatin structure and function [18].

The chromatin structure creates specific conditions for DNA damage and repair. Heterochromatin is known to suppress the generation of DSBs while preventing DNA repair [20]. In contrast, transcriptionally active sites in the genome are hot spots for DNA damage [21]. Importantly, DNA repair proteins modify histones, changing chromatin structure [22].

The histone marker for DSBs, γH2AX, is a phosphorylated form of histone H2AX that forms nuclear foci at sites of DNA breaks, helping in the recruitment of DNA repair proteins [23]. Poly(ADP-ribose) polymerase 1 (PARP1) is another essential biomarker of DNA damage and repair processes. PARP1 mediates DNA repair by catalyzing the formation of PAR chains, which help in DNA repair and chromatin compaction [24]. Both γH2AX and PARP1 are crucial for evaluating the efficacy of DNA-damaging compounds and provide valuable insights into the mechanisms underlying the DNA damage and repair deficiencies observed in neurodegenerative diseases.

Thus, dysfunctional cooperation between DNA damage/repair and chromatin structure may be a significant cause of neurodegeneration.

## Chromatin Structure and DNA Damage in the Context of Hallmark Proteins in Neurodegeneration

### TDP-43 Cooperates With Nucleosome Remodeling Enzymes

TAR DNA/RNA binding protein 43 (TDP-43) regulates transcription, mRNA processing, and possibly the cell cycle and apoptosis [25]. TDP-43 is also involved in the repair of the most cytotoxic type of DNA damage, double-stranded breaks (DSBs) [26, 27]. The presence of abnormal forms of TDP-43, such as TDP-43 mutations and aggregates in the central nervous system, is a common feature of several neurodegenerative diseases, such as amyotrophic lateral sclerosis (ALS), frontotemporal dementia (FTD), Alzheimer’s disease (AD), and limbic predominant age-related TDP-43 encephalopathy (LATE). In ALS, the TDP-43 mutants A315T, Q331K, and M337V induce DSBs, which are detectable by an increase in the phosphorylation of histone H2AX, called γH2AX (29, 30). Interestingly, TDP-43 knockdown via a specific siRNA prevents TDP-43 phosphorylation compared to that of the scramble control, suggesting that TDP-43 plays a role in histone modification during DNA damage and repair processes [26]. Importantly, TDP-43 and its dysfunctional neurodegenerative forms impact neuronal functions, which are governed by chromatin reorganization. TDP-43 participates in chromatin condensation/decondensation events, as decondensation of heterochromatic LINE retrotransposons and intergenic repeat chromatin due to loss of TDP-43 was observed [28]. The TDP-43 mutant A315T attenuates synaptic transmission, contributing to cognitive decline and motor deficits [29]. Similarly, depletion of TDP-43 enhances the acquisition of fear memory, decreases the short-term plasticity of intrinsic neuronal excitability, and slows the decay time of AMPAR-mediated miniature excitatory postsynaptic currents in transgenic rats [30]. Interestingly, DNA damage is also associated with cognitive processes [31].

Furthermore, TDP-43 interacts with a nucleosome remodeling enzyme of the SW12/SNF2 class within the ATP-dependent nucleosome remodeling superfamily (Chd1). This interaction is essential for the prevention of fly death, as the simultaneous knockdown of TDP-43 and Chd1 impairs the expression of heat shock genes and promotes lethality in flies [29]. In contrast, overexpression of Chd1 reversed these effects. This phenomenon also occurs in the mammalian cell line HEK393T. Under stress, TDP-43 strongly impairs histone clearance via the Hsp70 gene. Adding Chd1 restored histone clearance [29]. Moreover, cytoplasmic accumulation of TDP-43 is accompanied by a reduced level of Chd2 in the human cortex in patients with FTD (12).

Interestingly, the altered expression of genes involved in histone regulation, as well as DNA damage and repair, is correlated with pathological TDP-43 aggregation and TDP-43 nuclear loss [28]. Thus, a better understanding of the function of TDP-43 in chromatin modification during DNA damage and repair events could reveal an important disease mechanism of neurodegeneration.

### FUS Functions in the DNA Damage Response by Directly Interacting With Histone Modifiers

Fused in sarcoma (FUS) is a heterogeneous ribonucleoprotein (hnRNP) that belongs to the TET family of RNA-binding proteins (TAF15, EWS, and TLS). Variants in FUS genes are causative or risk factors for several neurodegenerative diseases, including ALS and FTD [32]. Like TDP-43, FUS functions in the repair of DSBs. FUS interacts with histone deacetylase 1 (HDAC1), a chromatin-modifying enzyme, to regulate the DNA damage response and to protect against DNA damage in neurons. However, for FUS proteins harboring familial ALS (fALS) mutations, R521C and P525L diminish this interaction, causing aberrant cell cycle re-entry and a defective DNA damage response [33]. In contrast, the overexpression of HDAC1 protects neurons from genotoxic agents [34]. Interestingly, the interaction between FUS and HDAC1 is detectable under physiological conditions in cortical neurons, but pharmacological induction of DNA damage with etoposide markedly enhances this interaction [35]. In addition, the immobilization of FUS to chromatin is sufficient to initiate the DNA damage response [34].

Another FUS-interacting protein, the FUS-interacting RNA binding protein RBM45 , is recruited to chromatin and to sites of DNA damage. Depletion of RBM45 results in excessive recruitment of HDAC1 to chromatin, impairing the repair of DSBs [35]. It is likely that RBM45 competes with HDAC1 for binding to FUS and therefore regulates the recruitment of HDAC1 to sites of DNA damage. Interestingly, the ALS-associated FUS mutation R521C preferentially interacts with RBM45 rather than with HDAC1 [35], likely disrupting DNA repair and contributing to neurodegeneration in individuals with ALS. At the level of synaptic function, the FUS-ALS-associated mutants R521C and P525L disrupt the formation of presynaptic active zones, subsequently reducing synaptic transmission and decreasing quantal size in *Drosophila* [36].

### SOD1 Dysfunction is Correlated With Histone Modification

Superoxide dismutase 1 (SOD1) is a free radical scavenging enzyme that forms a major component to guard against oxygen radical species produced during cellular metabolism. SOD1 is predominantly localized in the cytoplasm but is also localized within mitochondria, the nucleus, and the endoplasmic reticulum (ER) [37]. SOD1 mutants are present in familial ALS [37]. At the synapse level, SOD1 is localized at the pre- and post-synapse, while the ALS-associated mutant G93A SOD1 shows mislocalization in pre-synaptic terminals as well as at the post-synapse, impairing axonal transport and contributing to neuronal cell death [38, 39]. The expression of the mutant SOD1 G93A also decreases the formation of synaptophysin-positive presynaptic boutons [40].

SOD1 is also involved in both DNA damage and chromatin regulation. Motor neurons derived from transgenic SOD1 G93A mice are sensitive to glutamate toxicity, resulting in oxidative DNA damage, increased intracellular calcium levels, and mitochondrial dysfunction [41]. In peripheral blood mononuclear cells (PBMCs) derived from SOD1 ALS patients carrying various SOD1 mutations, analysis of differential expression between healthy controls and SOD1-mutant ALS patients revealed 635 significantly downregulated genes and 1406 significantly upregulated genes [42]. Among them, a gene encoding the synaptic protein synaptotagmin and synaptosomal-associated protein 25 (SNAP25), a biomarker for synaptic degradation, was central to the interaction network composed of the peak-associated genes exclusively found in SOD1 samples. Interestingly, H3K27me3, the repressive trimethylation of lysine 27 on histone H3 and a marker of facultative heterochromatin, was detected around the SNAP25 gene body. Importantly, SNAP25 is elevated in the early stages of Alzheimer’s disease (AD) [43]. Furthermore, H3K27me3 was found to be differentially expressed between PBMC samples derived from ALS patients and those derived from control individuals [42]. As heterochromatin is a transcriptionally inactive state of chromatin, enrichment of H2K27me3 may be a compensatory mechanism for increased SNAP25 expression in AD. It would be interesting to determine whether these events are accompanied by DNA damage induced by abnormal SOD1.

### Huntingtin-Dependent Chromatin Alteration and DNA Damage Are Significant Contributors to HD Pathogenesis

Huntingtin (HTT) is a soluble 3144-amino acid protein with the highest levels of expression in the CNS and testes. HTTs function in development and are involved in cell survival and intracellular transport. However, the physiological function of HTT is poorly understood [44]. HTT is expressed in the nucleus, ER, Golgi apparatus and neurites of neurons. Mutated HTT (mHTT) due to polyQ expansion is the major cause of Huntington’s disease (HD) [44].

DNA damage has been reported in post-mortem brain samples derived from HD patients, and the oxidative DNA damage marker 8-Oxo-dG is present in both nuclei and mitochondria [45, 46]. Interestingly, HD disrupts non-homologous end joining repair (NHEJ) through interaction with the Ku70 protein and leads to the accumulation of DSBs in primary neurons [47]. In contrast, the expression of exogenous Ku70 protects against abnormal behavior and pathological phenotypes in the R6/2 mouse model of HD [47]. mHTT may be toxic because of its synergistic effect on DNA repair and chromatin structure, as the histone-lysine methyltransferase dSETDB1 mediates mHTT-induced toxicity in a *Drosophila* model of HD. Nullifying dSETDB1 results in preserved external eye pigmentation and ommatidia compared to the GMR > p127Q line. In contrast, the expression of dSETDB1 slightly exacerbates mHTT (p127Q)-mediated eye degeneration and the loss of eye pigmentation [48]. In stratal HD cell lines, confocal microscopy revealed increased SETDB1 immunoreactivity in HDs (Q111/Q111), which was associated with increased h3K9me3-dependent heterochromatin condensation. These findings suggested that SETDB1/ESET is epigenetically regulated in a cell line model of HD. Furthermore, nogalamycin, a small-molecule chromatin- and DNA-binding drug, prevents this effect, decreasing H3K9me3 immunoreactivity and H3K9me3 chromatin condensation. A similar effect of nogalamycin on chromatin was observed in transgenic HD (R6/2) mice [48]. Nogalamycin also improved motor coordination and locomotor activity in these mice [48]. Interestingly, a biological network analysis revealed that H3K9me3-enriched epigenomes, which include topoisomerase 2 α, are involved in transcription, cellular protein metabolism processes, synapses, and DNA replication and repair processes in R6/2 mice. These findings indicate that H3K9me3 coordinates the regulation of multiple genes in cellular processes that may be involved in the pathogenesis of HD, including DNA damage and repair [48].

Altered chromatin accessibility and transcription are present in HD in an *in vitro* model of neural progenitor cells (NPCs), contributing to aberrant cell cycle re-entry and apoptosis throughout the progression from NPCs to astrocytes [49]. Moreover, genome-wide alterations in a number of epigenetic modifications, including DNA methylation and multiple histone modifications, are associated with HD, suggesting that mHTT causes complex epigenetic abnormalities and chromatin structural changes [50]. Like mouse studies [48], human HD genome-wide association studies (GWASs) revealed several modifier genes that affect the age of motor onset independently of the length of HTT CAG repeats, including genes involved in DNA repair and maintenance [51]. Interestingly, altered expression of genes involved in DNA repair was also found in human HD tissue samples with pathological lengths of CAG repeats [52]. In another study, 240 differentially methylated regions (DMRs) at promoters were identified in fully differentiated HD-hiPSCs. Among them, the promoter of a core component of the MILL/SET1 chromatin remodeling complex essential for H3K4me3, WD repeat-containing protein 5 (WDR5), was hypermethylated in HD-hiPSCs, causing its downregulation [53].

### α-Synuclein Modulates Chromatin and DNA Damage Repair

α-Synuclein is a highly soluble unfolded protein with multiple roles in synaptic vesicle trafficking, neurotransmitter release, and intracellular signaling events [54]. Epigenetic regulation is one of the mechanisms that controls α-synuclein expression. Abnormal intracellular deposition of toxic species of α-synuclein is associated with several neurodegenerative diseases, such as Parkinson’s disease, dementia with Lewy bodies, and multiple system atrophy [55].

α-Synuclein binds to DNA and histones to modulate DSB repair [56] and transcription [54]. α-Synuclein rapidly translocates to sites of laser-induced DNA damage in the nucleus of *in vivo* mouse brain cells, as well as in a mouse primary cortical neurons , where it may play a role in DNA repair [56]. It also plays a role in the regulation of histone modification [57]. α-Synuclein also interacts with the protein BRCA1-associated protein 1 (BAP1), which possesses ubiquitin C-terminal hydrolase activity (UCH) [58], the enzymatic activity of which is an important modulator of gene expression. Interestingly, breast cancer gene 1 protein BRCA1 functions in DNA damage repair, protein ubiquitination and chromatin remodeling [59].

Like FUS, α-synuclein regulates the function of HDACs. In several models, including cell lines and transgenic flies, α-synuclein restricts HDACs to the cytoplasm, and HDAC inhibition protects against the toxicity of α-synuclein [57]. Furthermore, α-synuclein interacts with the chief epigenetic eraser HDAC4, which is abundant in neurons [60].

### Impact of APP on Both DNA Damage and Chromatin Reorganization

β-Amyloid precursor protein (APP) initiates the formation of extracellular amyloid-β (Aβ) in AD [61]. The onset or progression of AD is associated with disrupted gene expression caused by changes in chromatin organization [62]. Interestingly, a subtle amount of DNA damage increases intraneuronal Aβ_1-42_ production in neurons cultured *in vitro* and in the cortex of the rodent brain [63]. APP participates in DNA repair through acetylation carried out by the acetyltransferase complex, which is an important process in the post-translational modification of chromatin [64].

The occurrence of DNA damage in AD is well established. DSBs and single-stranded breaks (SSBs) are present in the AD brain and at the early stage of disease development [5, 61, 63]. In parallel, aberrant epigenetic regulation and altered chromatin organization impacting synaptic plasticity and immune responses in AD have been reported [62]. Notably, epigenetic editing tools and small-molecule epidrugs targeting histone modifiers have emerged as potential therapeutic strategies [62, 63]. Interestingly, DNA damage and repair are directly linked to chromatin reorganization in AD. Acetylation, facilitated by the enzyme TIP60, plays a crucial role in reconstructing damaged DNA sites. The proteins APP and FE65, which are traditionally associated with transcriptional activation, also participate in DNA repair. These proteins bind to TIP60 and activate its enzymatic activity [64]. Overall, these findings reveal the relationship between DNA damage and chromatin reorganization in AD pathology.

### Tau Stabilizes Damaged DNA Ends

Tau is a microtubule-associated protein. However, in AD, these proteins undergo phosphorylation and form insoluble aggregates called neurofibrillary tangles (NFTs) [5]. Like Aβ, tau diminishes the ability of cells to maintain proper chromatin configuration [62]. Tau binds to DSBs [65]. However, the hyperphosphorylation of tau reduces this binding [66]. Interestingly, damaged chromatin may be stabilized and protected by the phosphorylated form of tau [67]. Tau, which is predominantly present at the nucleolus border, binds to the minor groove of DNA by creating a structure that resembles a histone binding [67]. However, as AD progresses, tau levels gradually decrease and reach their maximum limit when phosphorylated tau (AT100) is present only in intracellular NFTs during the late stages of AD [68]. As a result, global chromatin relaxation is induced by the departure of tau from neuronal nuclei. Tau that has departed from nuclei results in abnormal transcription of heterochromatin genes [69], euchromatin gene dysregulation [70], and other epigenetic alterations [71].

Tau exhibits a preference for specific genomic regions, particularly those positioned more than 5000 base pairs from transcription start sites, as identified through chromatin immunoprecipitation with DNA microarray (ChIP-on-chip) techniques [72]. An AG-rich DNA motif is recurrent within tau-interacting regions, with approximately 30% of these regions overlapping with DNA sequences coding for long non-coding RNAs. Notably, the neurological processes affected in AD are enhanced among tau-interacting regions [72]. The plasticity of the interaction of tau with genes is highlighted under heat stress conditions, suggesting a potential regulatory role in gene expression [73]. Additionally, DNA repair nuclear protein BRCA1 colocalizes with  tau aggregates in various tauopathies, including AD, FTD, and progressive supranuclear palsy [74]. The tau protein has been found to regulate neuronal pericentromeric heterochromatin, influencing the structure and function of H3K9me3 and other proteins [75].

Furthermore, tau is a nucleolar protein that associates with the transcription termination factor I-interacting protein 5 (TIP5), potentially contributing to the regulation of rDNA transcription and heterochromatin stability [76]. The protective role of tau in maintaining neuronal DNA and RNA integrity has been emphasized, with tau deficiency leading to increased DNA damage under normal and heat stress conditions [73, 76]. Remarkably, tau stabilizes double-stranded DNA structures, slowing denaturation and protecting DNA from free radical damage [65]. Tau interacts with DNA by binding to double-stranded DNA in a charge-dependent manner, and the process is rapid and reversible [65, 77].

## Conclusions

This review extends the understanding of the role of DNA damage in neurons and highlights its significance for promoting neurodegeneration. Given that DNA damage impacts neuronal function, the role of DNA damage in neurons goes beyond simple neuronal death and encourages revaluation of the role of DNA damage in neuronal function. The proteins involved in DNA damage and repair can directly modulate chromatin, but it is also possible that due to increased DNA damage in the progression of neurodegenerative diseases, their expression increases, and thus, they interact with chromatin. The interplay between DNA damage and chromatin rearrangement requires further studies to determine the detailed mechanisms underlying the role of DNA damage/chromatin in neurodegeneration. This relationship across various neurodegenerative diseases is associated with the pathogenesis of these diseases and provides hope for the development of effective therapies. Illustration 1 and Table 1 summarize the dual roles of the described proteins in DNA damage and chromatin rearrangement.

**Illustration 1:**
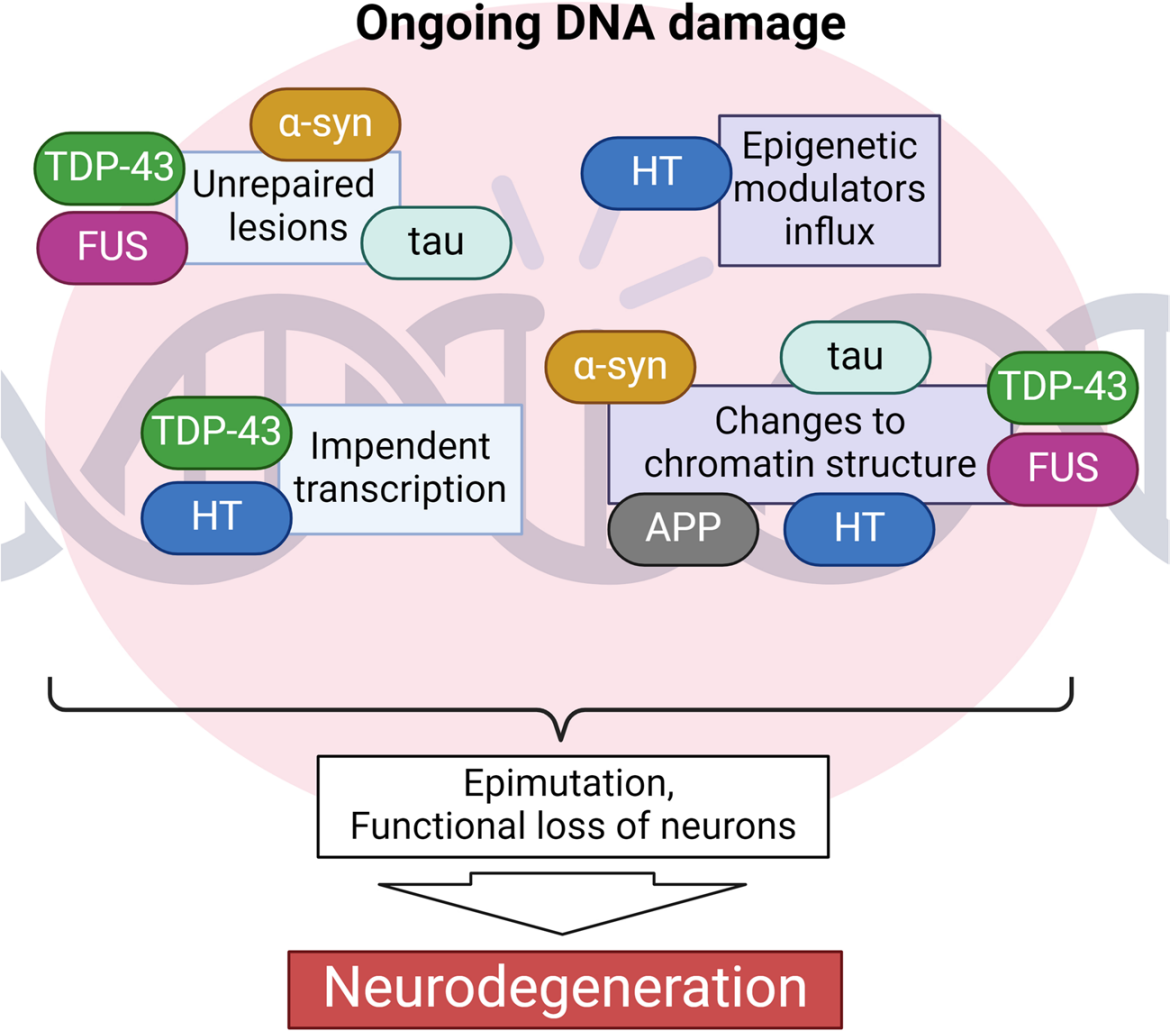
A simplified illustration of the interplay between DNA damage and chromatin organization in neurodegeneration. DNA damage and chromatin reorganization are inseparable processes. A number of proteins associated with various neurodegenerative diseases impact both processes during ongoing DNA damage, binding together distinct disease mechanisms. *TDP-43, TAR DNA/RNA binding protein 43; FUS, fused in sarcoma; HT, Huntingtin; α-syn, α-synuclein; APP, β-amyloid precursor protein; tau, a microtubule-associated protein*

**Table 1:** Summary of the link between DNA damage and chromatin re-organization in the dysfunction of hallmark proteins of neurodegeneration

Protein	Associated neurodegenerative disease	DNA damage/chromatin link
TDP-43 (TAR DNA/RNA binding protein 43)	• Amyotrophic lateral sclerosis • Frontotemporal dementia • Alzheimer’s disease • Limbic predominant age-related TDP-43 encephalopathy	• TDP-43 knockdown interferes with phosphorylation of histone H2AX triggered by DNA damage (26) • Pathological TDP-43 aggregation is accompanied with changes in gene expression responsible for both, histone regulation, and DNA repair (28)
FUS (fused in sarcoma)	• Amyotrophic lateral sclerosis • Frontotemporal dementia	• Regulates DNA damage response through binding to chromatin-modifying enzyme, HDAC1 (33)
SOD1 (superoxide dismutase 1)	• Amyotrophic lateral sclerosis • Parkinson’s disease	*Understudied*
Huntingtin	• Huntington’s disease	• In transgenic HD mouse, H3K9me3-enriched epigenomes is involved in DNA repair processes (48). • In human HD, DNA damage gene modifiers, which affect HD onset were reported (51).
α-Synuclein	• Parkinson’s disease • Dementia with Lewy body • Multiple system atrophy	• It modulates DSB repair by binding to DNA and histones (55). • Interacts with BAP1 protein, which associates with BRCA1 protein, involved in chromatin re-modeling and DNA damage (57, 58)
APP (β-amyloid precursor protein)	• Alzheimer’s disease	• Participates in DNA repair through modulation of post-translational modification of chromatin (59) • Involved in reconstruction of DNA after DNA damage (63)
Tau	• Alzheimer’s disease • Frontotemporal dementia • Progressive supranuclear palsy	• Binds to DNA damage (67) and diminishes cell capability to proper configuration of chromatin (61) • Stabilizes double-stranded DNA structure to protect it from oxidative DNA damage (6)

Current treatments for neurodegenerative diseases are mostly symptomatic, with disease symptoms diminishing for a limited period. The importance of chromatin rearrangement in DNA damage and its association with other critical processes, such as aberrant synaptic plasticity and gene expression, make chromatin rearrangement in the context of DNA damage an attractive therapeutic target. Importantly, such epigenetic approaches could include reuse of HDAC inhibitors, which are already approved for cancer treatment and include sodium phenylbutyrate, valproic acid, and vorinostat [78, 79]. Another approach could rely on microRNA-based therapeutics to compensate for microRNAs, which are dysregulated in neurodegeneration; these include miR-9, miR-29, miR-15, miR-34, and miR-21-5p [80, 81].
